# Prevalence and factors associated with fatigue in patients with ulcerative colitis in China: a cross-sectional study

**DOI:** 10.1186/s12876-022-02357-z

**Published:** 2022-06-03

**Authors:** Feng Xu, Jingyi Hu, Qian Yang, Yuejin Ji, Cheng Cheng, Lei Zhu, Hong Shen

**Affiliations:** Affiliated Hospital of Nanjing University of Chinese Medicine (Jiangsu Province Hospital of Chinese Medicine), Nanjing, 210029 Jiangsu Province China

**Keywords:** Ulcerative colitis, Fatigue, Risk factors, Disease activity, China

## Abstract

**Background:**

Fatigue is one of the most common symptoms reported by patients with ulcerative colitis (UC), while it has not been fully recognized and taken seriously in clinical practice. We aimed to investigate the prevalence of fatigue in patients with UC and identify the factors associated with fatigue and its severity in China.

**Methods:**

A cross-sectional study was conducted in Affiliated Hospital of Nanjing University of Chinese Medicine from May 2020 to February 2021. Demographic and clinical characteristics were collected. Fatigue was evaluated with the Fatigue Severity Scale and the Multidimensional Fatigue Inventory. The Hospital Anxiety and Depression Scale, the Pittsburgh Sleep Index Scale and the Malnutrition Universal Screening Tool were respectively used to evaluate the anxiety, depression, sleep disturbance and nutritional risk of patients with UC.

**Results:**

A total of 220 UC patients were enrolled in this study. The prevalence of fatigue in patients was 61.8%, of which in patients with disease activity was 68.2%, and in patients in remission was 40.0%. Univariate analysis indicated that the Montreal classification, disease activity, anemia, anxiety, depression, sleep disturbance and high nutritional risk were the factors associated with fatigue in Patients with UC. Multivariate logistic regression analysis showed that the Montreal classification (E3: E1, OR = 2.665, 95% CI = 1.134–6.216), disease activity (OR = 2.157, 95% CI = 1.055–4.410) and anxiety (OR = 2.867, 95% CI = 1.154–7.126) were related to an increased risk of fatigue. Disease activity (RC = 0.240, 95% CI = 0.193–0.674) and anxiety (RC = 0.181, 95% CI = 0.000–0.151) were associated with severity of fatigue.

**Conclusions:**

This study demonstrated that the prevalence of fatigue among UC patients in China. The Montreal classification, disease activity and anxiety are associated with an increased risk of fatigue.

## Background

Ulcerative colitis (UC) is a kind of chronic inflammatory disease affecting the mucosal of colon and rectum [1, 2]. Diarrhea, blood stool and abdominal pain have been considered to be the common clinical manifestations of UC [3]. How to control these symptoms has been extensively studied. With the promotion and application of advanced therapies such as anti-tumor necrosis factor (TNF) therapy, anti-adhesion therapy, anti-interleukin (IL)-12/IL-23p40 therapy and Janus kinase (JAK) inhibitor therapy in recent years, the treatment goals of UC have been shifted from traditional symptom relief and mucosal healing to histological relief and the improvement of quality of life in patients [4–6].

Fatigue is considered to be a subjective discomfort, accompanied by an unrelievable lack of energy or feeling of exhaustion, which reduces patient’s ability of work physically and mentally [7, 8]. Fatigue has been identified as one of the most important clinical symptoms in patients with Inflammatory bowel disease (IBD) and a clinical problem that needs to be solved urgently [9]. Relevant studies have shown that the prevalence of fatigue in IBD patients is fivefold higher than that described in the general population [10]. The prevalence of this symptom is as high as 52% in the active phase in UC patients [11]. It is reported that 30–48% of patients with UC in remission still suffered from fatigue [12, 13]. High prevalence of fatigue in UC emphases the importance of additional efforts to assess and exam fatigue to improve the care and quality of life for patients. A variety of factors, including disease activity, anemia, anxiety, depression and sleep disturbance are particularly prominent in UC patients with fatigue, which have a negative impact on the quality of life and work efficiency [11, 14–16]. Despite this, fatigue in UC patients tend to be neglected and is poorly managed in clinical diagnosis and treatment [17]. In addition, only few researches have focused on the factors associated with its occurrence and its severity in patients with UC, and there are still many controversial aspects.

The aims of the present study were to investigate the prevalence of fatigue in patients with UC and identify the factors associated with fatigue or its severity in China.

## Materials and methods

### Study population

This cross-sectional study was conducted at the Affiliated Hospital of Nanjing University of Chinese Medicine from May 2020 to February 2021. The criteria for inclusion were verified diagnosis of UC in patients who were at least 18 years old and signed informed consent. The diagnostic criteria for UC were based on the Chinese consensus on diagnosis and treatment in IBD [18]. Patients with severe cardiovascular disease, liver, kidney, blood system disease, diagnosed with cognitive impairment or psychiatric disease (except anxiety and depression) and unable to express their condition accurately were excluded.

### Data collection

The information of demographic and disease characteristics of UC patients were collected via a unified questionnaire. The variables included age, gender, level of education, employment status, only child or not, disease duration, the Montreal classification, disease activity, extra-intestinal manifestations (EIMs), complication, medication have been used, IBD related surgery, smoking status, drinking status and body mass index (BMI) were collected from clinical records.

Five self-administered questionnaires were completed by the patients to evaluate the severity and different dimensions of fatigue, anxiety, depression and sleep disorders. Nutritional risk of patients was evaluated by the Malnutrition Universal Screening Tool (MUST).

### Ethics

The study was conducted according to the principles of Declaration of Helsinki. The ethics committee of the Affiliated Hospital of Nanjing University of Chinese Medicine approved this study (2020NL-091-02). Written informed consent was obtained from all patients.

### Definitions

Disease extent were classified by the Montreal classification. E1 represents Proctitis, E2 represents Left side colitis and E3 represents Extensive colitis [19]. Disease activity and severity of UC were assessed according to Mayo score and Truelove and Witts criteria [20, 21]. Anemia was defined as hemoglobin < 12 g/dL for males and < 11 g/dL for females. Underweight was defined as BMI < 18.5 kg/m^2^; normal weight was defined as 18.5 kg/m^2^ ≤ BMI < 24.0 kg/m^2^; overweight was defined as 24.0 kg/m^2^ ≤ BMI < 28.0 kg/m^2^ and obesity was defined as BMI ≥ 28.0 kg/m^2^.

### Questionnaires

The Fatigue Severity Scale (FSS) is a standardized scale containing 9 items. Each item is scored from 1 (completely disagree) to 7 (completely agree). FSS score is the mean score of all items. In accordance with previous studies, fatigue was defined as a mean FSS score > 4 points [22, 23].

The Multidimensional Fatigue Inventory (MFI) is a fatigue assessment tool which contained 20 items. MFI was designed to assess the five dimensions of fatigue, including general fatigue, physical fatigue, mental fatigue, decreased motivation and reduced activity. Each dimension includes 4 items, ranging from 4 to 20 scores. Higher scores indicate more severe fatigue [24]. Although it is the most commonly used scale for evaluating fatigue in IBD, it lacks a clear cutoff score to define fatigue.

Anxiety and depression were assessed with the Hospital Anxiety and Depression Scale (HADS). The scale consists of two subscales, the Hospital Anxiety Scale (HAS) and the Hospital Depression Scale (HDS), both with a total score of 0–21. In previous studies, 8 is usually divided into the cutoff score of anxiety and depression [25, 26]. The analysis of research data in China showed that a cutoff value of 9 (anxiety/depression score ≥ 9) is more reliable [27]. This study used the cutoff value of 9 as the critical value for screening anxiety/depression.

The Pittsburgh Sleep Quality Index (PSQI) was formulated by Dr. Buysse at University of Pittsburgh in 1989. It was a widely used sleep assessment tool in clinical practice and was often used to assess the sleep quality of IBD patients. The scale is divided into 7 parts, including sleep quality, sleep latency, sleep duration, habitual sleep efficiency, sleep disturbance, use of sleeping medication, and daytime dysfunction. Each part is scored from 0 to 3, and the total score ranges from 0 to 21. The total score > 5 indicates the presence of sleep disorders [28].

Malnutrition Universal Screening Tool (MUST) is a commonly used tool to access the nutritional status of patients with IBD, including BMI, weight loss in the last 3–6 months and the impact of acute disease [29, 30]. Each item is scored from 0 to 2 and the total score ranges from 0 to 6. A total score of 0 indicates low nutritional risk, a score of 1 indicates a medium nutritional risk and a score of ≥ 2 indicates high nutritional risk.

### Statistical analysis

The quantitative variables were expressed as the mean and standard deviation (SD) or the median and interquartile range (IQR), depending on whether the variables were normally distributed or not. Categorical variables were expressed as number of patients and percentage. Univariate analysis was compared using the *χ*^2^ test and the Fisher exact test or the Wilcoxon rank-sum test according to the distribution of values. Correlations between the score of FSS and MFI, HADS, PSQI and MUST were measured with the Pearson correlation coefficients.

The variables that were statistically significant in univariate analysis were enrolled in multivariate analysis. The results were expressed as odds ratios (ORs) with their corresponding 95% confidence intervals (95%CIs). In addition, multiple linear regression analysis was performed to identify whether the demographic and disease characteristics of this cohort associated with the severity of fatigue. The Statistic Package for Social Science 25 (SPSS Inc., Chicago, IL, United States) was used for statistical analyses. Statistical significance was set at *P* < 0.05.

## Results

### Study population

A total of 220 UC patients were included in this study and were classified as having fatigue (n = 136) or none fatigue (n = 84) according to the FSS score reported by patients. The participants had a median age of 45 (IQR: 33–56) years. In terms of the duration of disease, the participants had a median of 3.9 (IQR: 1.3–7.9) years. A total of 77.3% (n = 170) of UC patients were in active disease. 50% (n = 110) of the patients had an education level of high school or below. Among these UC patients, only child accounted for 31.8% (n = 70). There were 68.6% (n = 151) patients had a full time or part time job. The drugs had been used by patients were mainly 5-aminosalicylic acid (5-ASA) and corticosteroids, which accounted for 98.2% (n = 216) and 25.9% (n = 57) of the total number of patients respectively. The demographic and disease characteristics of UC patients are summarized in Table 1.

**Table 1:** Demographic and disease characteristics in patients with ulcerative colitis (UC) with fatigue and none fatigue

Variables	Total (n = 220)	Fatigue (n = 136)	None fatigue(n = 84)	*P* value
*Gender, n (%)*				
Male	131 (59.5)	80 (58.8)	51 (60.7)	0.781
Female	89 (40.5)	56 (41.2)	33 (39.3)	
Age, year, median (IQR)	45 (33–56)	44 (32–56)	48 (35–57)	0.177
*Education, n (%)*				
Master degree or above	9 (4.1)	5(3.7)	4(4.8)	0.861
Undergraduate	62 (28.2)	37 (27.2)	25 (29.8)	
Junior college	39 (17.7)	23 (16.9)	16 (19.0)	
Senior high school or below	110 (50.0)	71 (52.2)	39 (46.4)	
*Employment status, n (%)*				
Full time or part time	151 (68.6)	91 (66.9)	60 (71.4)	0.134
No work	15 (6.8)	13 (9.6)	2 (2.4)	
Retired	37 (16.8)	20 (14.7)	17 (20.2)	
Other	17 (7.7)	12 (8.8)	5 (6.0)	
*Only child, n (%)*				
Yes	70 (31.8)	47(34.6)	23(27.4)	0.267
No	150 (68.2)	89(65.4)	61 (72.6)	
Disease duration, year, median (IQR)	3.9 (1.3–7.9)	4.0 (1.4–8.0)	3.8 (1.1–6.7)	0.649
*Montreal classification, n (%)*				
E1	39 (17.7)	16 (11.8)	23 (27.4)	< 0.001
E2	66 (30.0)	33 (24.3)	33 (39.3)	
E3	115 (52.3)	87 (64.0)	28 (33.3)	
*Disease activity, n (%)*				
Remission	50 (22.7)	20 (14.7)	30 (35.7)	< 0.001
Mild	88 (40.0)	47 (34.6)	41 (48.8)	
Moderate	59 (26.8)	47 (34.6)	12 (14.3)	
Severe	23 (10.5)	22 (16.2)	1 (1.19)	
*EIM, n (%)*				
Yes	65 (29.5)	38 (27.9)	27 (32.1)	0.507
No	155 (70.5)	98 (72.1)	57 (67.9)	
*Complications, n (%)*				
Yes	12 (5.5)	5 (3.68)	7 (8.3)	0.241
No	208 (94.5)	131 (96.3)	77 (91.7)	
*Medication have been used, n (%)*				
5-ASA	216 (98.2)	133 (97.8)	83 (98.8)	0.686
Immunosuppressants	10 (4.5)	6 (4.4)	4 (4.8)	
Corticosteroids	57 (25.9)	40 (29.4)	17 (20.2)	
Biologic treatment	8 (3.6)	5 (3.7)	3 (3.6)	
*IBD related surgery, n (%)*				
Yes	10 (4.5)	8 (5.9)	2 (2.4)	0.226
No	210 (95.5)	128 (94.1)	82 (97.6)	
*Anemia*				
Yes	49 (22.3)	38 (77.6)	11 (22.5)	0.01
No	171(77.7)	98 (57.3)	73 (42.7)	
*Smoking status, n (%)*				
Yes	42(19.1)	26 (19.1)	16 (19.0)	0.99
No	178(80.9)	110 (80.9)	68 (81.0)	
*Drinking status, n (%)*				
Yes	185 (80.1)	21 (15.4)	14 (16.7)	0.809
No	35 (15.9)	115 (84.6)	70 (83.3)	
*BMI, n (%)*				
Thinnish	34 (15.5)	24 (17.6)	10 (11.9)	0.32
Normal	136 (61.8)	79 (58.1)	57 (67.9)	
Overweight or Obesity	50 (22.7)	33 (24.3)	17 (20.2)	
FSS, median (IQR)	4.4 (3.3–5.9)	5.4 (4.6–6.3)	2.7 (2.0–3.3)	< 0.001
MFI, median (IQR)	61 (48–73)	68.5 (60–78)	48 (37.3–58)	< 0.001
HAS, median (IQR)	5 (3–8)	7 (4–10)	4 (2–6)	< 0.001
HDS, median (IQR)	6 (3–9)	7 (4–10)	5 (2.25–7)	< 0.001
PSQI, median (IQR)	6 (4–9)	7 (5–10)	5 (3–8)	0.001
MUST, median (IQR)	1 (0–2)	1 (0–2)	0 (0–1)	0.001

*IQR* indicates interquartile range, E1, Proctitis; E2, Left side colitis; E3, Extensive colitis; *EIM* extra-intestinal manifestations; *5-ASA* 5-aminosalisylic acid, *IBD* inflammatory bowel disease, *BMI* body mass index, *HAS* hospital anxiety scale, *HDS* hospital depression scale, *PSQI* pittsburgh sleep quality index, *MUST* malnutrition universal screening tool

### Prevalence and score of fatigue

**Fig. 1:**
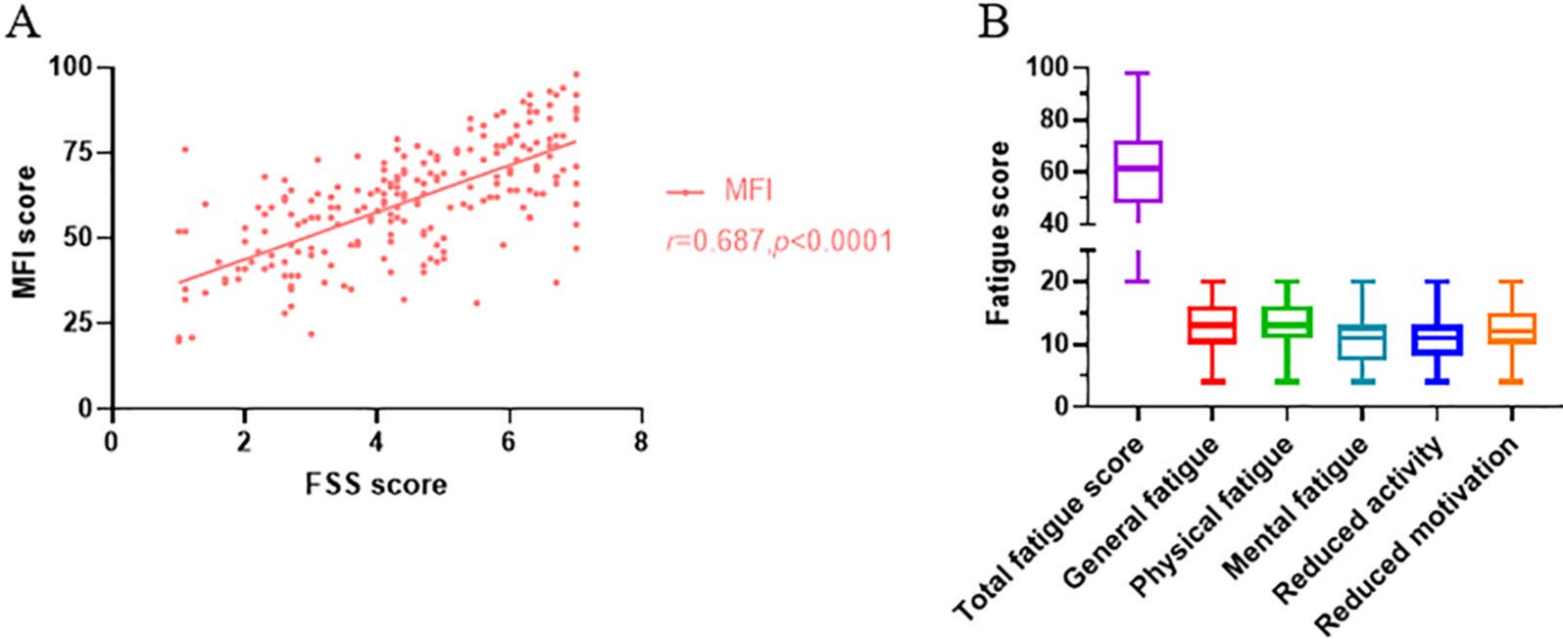
Fatigue score in ulcerative colitis patients. **A** Correlation between FSS score and MFI score in patients with UC. Pearson *r* = 0.687; *P* < 0.0001. **B** The fatigue score of different MFI domains

The prevalence of fatigue in patients with UC, defined as a FSS score > 4, was 61.8% in the total study population. The prevalence of fatigue in patients with active disease was 68.2%, which was remarkably high compared with those in remission (40.0%). The score of MFI showed a positive correlation with FSS score (*r* = 0.687; *P* < 0.0001) (Fig. 1A). The median FSS score of UC patients was 4.4(IQR: 3.0–5.9). The median total score of MFI was 61 (48–73). The scores of different MFI domains were general fatigue: 13 (10–16); physical fatigue 13 (11–16); mental fatigue11 (7.25–13); reduced activity 11 (8.25–13) and reduced motivation 12 (10–15) (Fig. 1B).

### Factors associated with the presence and severity of fatigue

**Fig. 2:**
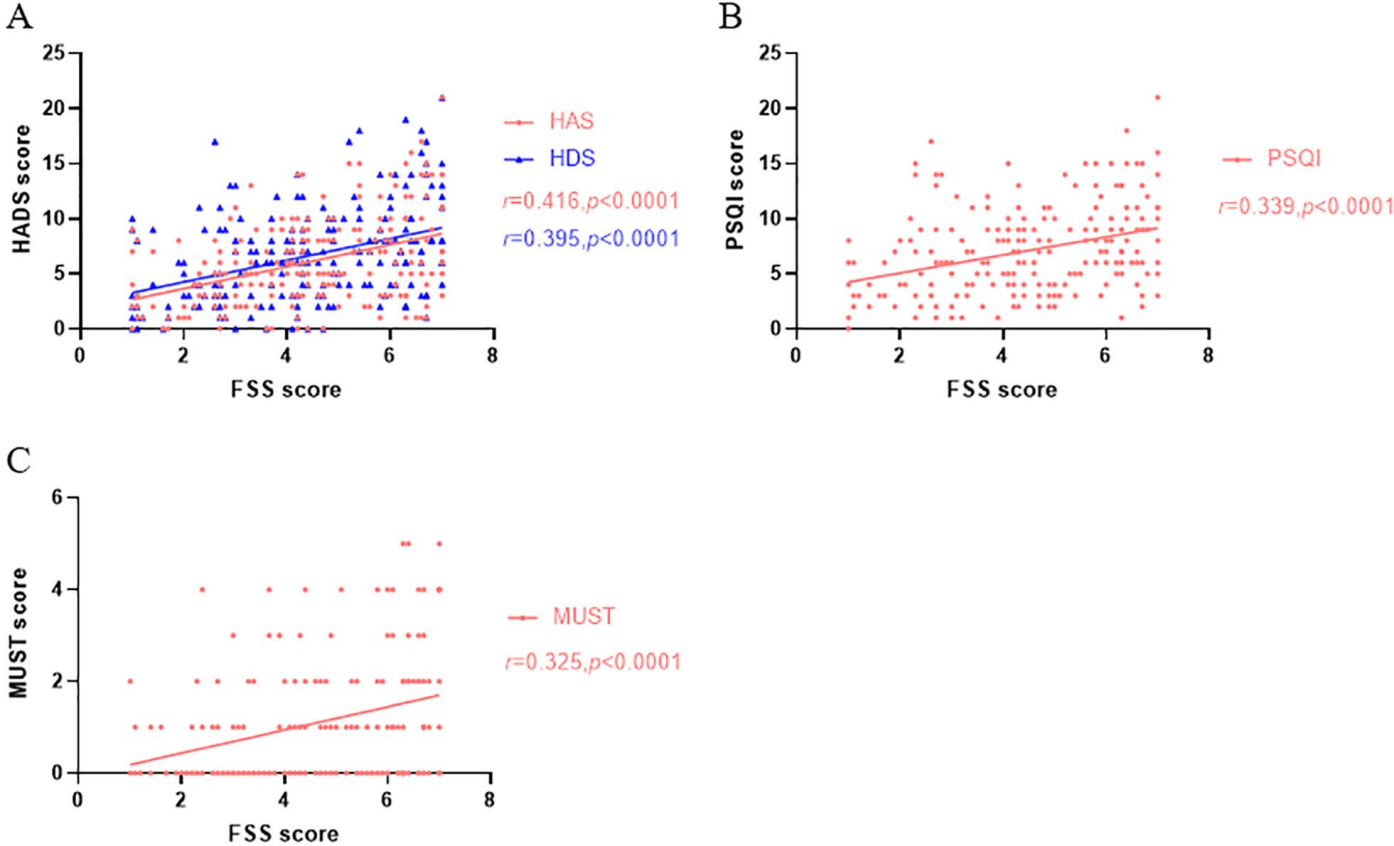
Correlation between FSS and HADS, PSQI and MUST score in patients with UC. **A** Correlation between FSS score and HADS score. Correlation between FSS score and HAS score: Pearson *r* = 0.416, *P* < 0.0001; Correlation between FSS score and HDS score: Pearson *r* = 0.395, *P* < 0.0001. **B** Correlation between FSS score and PSQI score: Pearson *r* = 0.339, *P* < 0.0001. **C** Correlation between FSS score and MUST score: Pearson *r* = 0.325, *P* < 0.0001

The univariate analysis indicated that the Montreal classification, disease activity, anxiety, depression, sleep disturbance and risk of nutrition were the factors associated with fatigue in ulcerative colitis patients (Table 1). Further statistical analysis showed that FSS was positively correlated with HAS (*r* = 0.416, *P* < 0.0001), HDS (*r* = 0.395, *P* < 0.0001), PSQI (*r* = 0.339, *P* < 0.0001) and MUST(*r* = 0.325, *P* < 0.0001) (Fig. 2).

Multivariate logistic regression analysis indicated that the Montreal classification (OR = 2.655, 95% CI:1.134–6.216), disease activity (OR = 2.157, 95% CI:1.055–4.410) and anxiety (OR = 2.867, 95% CI:1.154–7.216) were related to a higher risk of presence of fatigue in UC patients (Table 2).

**Table 2:** Factors associated with presence of fatigue in multivariate logistic regression analysis

Factors	OR	95% CI	*P*
Montreal classification (E2:E1)	1.146	0.484–2.713	0.757
Montreal classification (E3:E1)	2.655	1.134–6.216	0.024
Disease activity	2.157	1.055–4.410	0.035
Nutritional risk (medium:low)	1.430	0.651–3.140	0.373
Nutritional risk (high:low)	1.420	0.663–3.041	0.367
Anemia	1.700	0.744–3.883	0.208
Anxiety	2.867	1.154–7.126	0.023
Depression	1.529	0.695–3.363	0.291
Sleep disturbance	1.339	0.712–2.519	0.365

*OR* indicates Odds ratio, *CI* confidence interval, *E3* indicates extensive colitis, E1, Proctitis

Multiple linear regression analysis showed that increased disease activity (RC = 0.240, 95% CI: 0.193–0.674) and anxiety (RC = 0.181, 95% CI: 0.000–0.151) were independently associated with the severity of fatigue. These factors explained 31.2% of the variance (Table 3).

**Table 3:** Factors associated with severity of fatigue in the linear regression analysis

Factors	RC	95% CI	*P*	R^2^
Montreal classification	0.083	− 0.087 to 0.450	0.183	31.2
Disease activity	0.240	0.193 to 0.674	< 0.001	
Nutritional risk	0.084	− 0.059 to 0.277	0.203	
Anemia	0.071	− 0.211 to 0.774	0.260	
Anxiety	0.181	0.000 to 0.151	0.049	
Depression	0.102	− 0.032 to 0.114	0.268	
Sleep disturbance	0.083	− 0.021 to 0.091	0.225	

*RC* indicates standardized regression coefficient, *CI* indicates confidence interval, R^2^ coefficient of determination

## Discussion

In this cross-sectional study, we confirmed that fatigue is considerably high in UC patients in China. The prevalence of fatigue was 61.8%. 68.2% patients with disease activity and 40.0% in the remission stage reported fatigue. The major factors related to the presence of fatigue in UC patients were the Montreal classification, disease activity and anxiety. Furthermore, disease activity and anxiety were associated with the severity of fatigue. Fatigue was not associated with female gender, education level, disease duration, complication and extra-intestinal manifestations and IBD related surgery. To our knowledge, this is one of the few researches on the prevalence and factors associated with fatigue in patients with UC in China.

Fatigue was described as a complex and multidimensional symptom [17, 31]. Patients suffer from chronic inflammatory disease such as Rheumatoid arthritis, Systemic lupus erythematosus and Sjogren’s syndrome had a higher risk of fatigue [32–35]. UC patients were no exception, especially those in active phase [36, 37]. Our research showed that disease activity was an independent risk factor for fatigue and related to the severity of fatigue, which is in accordance with previous studies [11, 38, 39]. In contrast, some studies did not find any association between disease activity and the presence of fatigue nor its severity [10, 40]. It is possible that the assessment of fatigue was not identical among various studies, which influenced the results.

In present study, the Montreal classification, reflecting disease extent of ulcerative colitis, was associated with the presence of fatigue. UC patients classified as extensive colitis were more likely to report fatigue than those classified as Proctitis. This finding is disagreement with previous studies [41, 42]. The possible reasons for the discrepancies were that the number of patients included in previous studies was not large enough to observe the correlation between the Montreal classification and fatigue. Moreover, differences in statistical methods may also cause the relationship between the Montreal classification and fatigue to be ignored. As far as we know, most previous studies only performed univariate analysis to perceive their relationship.

Fatigue is an extremely common and disabling symptom complained among patients with chronic disease, including patients with UC [7, 36]. Psychological comorbidity such as anxiety, depression and sleep impairment were considered to be related to fatigue in IBD [14, 43–45]. In our study, anxiety was the risk factor of fatigue and related to the severity of fatigue, which is in line with existing studies [10, 46]. The present study indicated weak to moderate correlation between fatigue and depression, but no statistical difference was fond in the regression analysis model. Coincidentally, although sleep disturbance is a related factor of fatigue in univariate analysis, there is no correlation between sleep disturbance and prevalence of fatigue or its severity in regression analysis. Some studies had reached the opposite conclusion [12, 14, 45]. A possible explanation for these conflicting data might be different definitions of depression and sleep disturbance. Secondly, different scales used to assess depression and sleep disturbance may cause conflicting results to some extent. Last but not the least, the differences in social conditions between China and western countries could not be ignored. Alcohol and drug abuse were considered to be associated with emotional distress and poor sleep quality in western countries, which contribute to fatigue in IBD patients. Influenced by traditional Chinese culture, Chinese people consumed less alcohol and had less drug abuse than people from western countries. Therefore, our study showed that depression and sleep disturbance in patients were not as serious as compared to related studies in western countries [47].

In our study, approximately a quarter of the patients had anemia. Univariate analysis confirmed that patients with anemia had a higher prevalence of fatigue than those without anemia. As expected from previous studies, anemia was the significant predictors of fatigue in IBD [48, 49]. Nevertheless, we did not find a positive correlation between anemia and the presence of fatigue or its severity in multivariate analysis. The results were in agreement with some other studies [10, 50, 51]. About two-thirds of the patients included in present study were in remission or mild activity, indicating that the bloody stools and other clinical symptoms of UC patient were not serious and persistent. It allows patients to adapt to this slow and mild change without feeling fatigue. Furthermore, different statistical methods performed among these studies were also responsible for these conflicting results.

Propositions on the relationship between fatigue and nutritional deficiency was inconsistent currently. Several studies confirmed that nutritional deficiency, including but not limited to Vitamin B12 and Iron deficiency, could contribute to fatigue [11, 52]. By contrast, additional studies have suggested no difference in IBD patients with or without fatigue in level of Vitamin D or Vitamin B12 [41, 51, 53]. Our study demonstrated that the degree of nutritional risk was related to fatigue in univariate analysis. The same as depression, sleep disorders, and anemia, there is no correlation between nutritional risk and fatigue in multivariate analysis. Most of the included patients had mild disease and the nutritional risk was low to moderate. Therefore, the relationship between fatigue and nutritional risk tends to be overlooked. More researches need to be conducted to confirm the relationship between fatigue and nutritional risk.

In our study, education level, disease duration, complication and extra-intestinal manifestations were unrelated to fatigue in UC patients. These findings were in line with literature data and not unexpected [46, 54]. Female gender had previously been found to be a predictor of UC patients reported fatigued [11, 48, 55]. In some past studies, IBD related surgery were related to fatigue [11, 56]. However, some other studies have reached the opposite conclusions [46, 51, 54]. Our results did not identify any significant association between fatigue and gender or IBD related surgery. The number of patients included and the method evaluating fatigue vary from study to study. It may be the cause of these differences.

Previously, the causes of fatigue associated with UC were identified to a limited extent. Although several independent factors associated with severity of fatigue were identified in this study, these factors only explained 31.2% of the variance. This reaffirmed the complexity and multifactorial etiology of fatigue. There were many potential factors that had not been investigated. Other contributors to fatigue in patients with UC may include irritable bowel syndrome (IBS), chronic pain syndrome, poor quality of life, high disability and decreased physical function. A study conducted in Norway indicated that coexisting IBS-like symptoms might be associated with increased levels of fatigue among IBD patients in remission [57]. Another cross-sectional study performed in United Kingdom (UK) found that IBD patients with IBS-type symptoms reported higher fatigue severity [15]. Besides fatigue, pain is one of the major symptoms that exists in IBD. A survey conducted among Norwegian IBD patients showed that patients reporting fatigue had significantly higher pain intensity scores compared to those without fatigue [58]. Previous research had confirmed that fatigue was independently associated with an impaired Inflammatory Bowel Disease Questionnaire (IBDQ) in UC patients [48]. A French nationwide survey reported that fatigue was the most frequent disabling symptoms at work and seriously impacted their working life. In addition, 72% of the respondents already had or wanted to get the disabled worker status [59]. Moreover, fatigue produced numerous negative impacts on daily activities in patients with IBD, including but not limited to decreased physical function, absence from work, reduced work productivity and impaired work activity [56, 59].

Fatigue was listed as one of the official symptoms of Coronavirus disease 2019 (COVID-19) in UK [60]. Coincidentally, our study was performed during the global COVID-19 pandemic, which may have influenced the results. Although China was one of the first countries to suffer from the outbreak of COVID-19, the pandemic was quickly controlled with very few sporadic cases nationwide. Furthermore, all patients who came to the hospital were advised to perform a COVID-19 nucleic acid test to rule out the viral infection. Therefore, the impact of COVID-19 on the results of this study was extremely limited.

This study presents some limitations. On one hand, the cross-sectional study, which makes it impossible to observe fatigue in a longitudinal perspective. On the other hand, the sample size is too small to catch more variables related to fatigue in UC patients on multivariate analysis. These variables need to be verified in a larger number of patients in the future.

In conclusion, the prevalence of fatigue in UC patients is remarkably high. A considerable percentage of patients with active disease as well as patients who fulfilled the criteria for deep remission suffer from fatigue. Univariate analysis indicated that the Montreal classification, disease activity, anemia, anxiety, depression, sleep disturbance and high nutritional risk were the factors associated with fatigue in Patients with UC. In multivariate analysis, the Montreal classification, disease activity and anxiety were associated with an increased risk of fatigue. Its severity was associated with disease activity and anxiety. The results of this study provide a scientific basis for understanding and managing fatigue in patients with UC.

## Data Availability

All dataset used for this study are available from corresponding author on reasonable request.

## Abbreviations

UC: Ulcerative colitis
TNF: Tumor necrosis factor
IL: Interleukin
JAK: Janus kinase
IBD: Inflammatory bowel disease
FSS: Fatigue severity scale
MFI: Multidimensional fatigue inventory
HADS: Hospital anxiety and depression scale
HAS: Hospital anxiety scale
HDS: Hospital depression scale
PSQI: Pittsburgh sleep index scale
MUST: Malnutrition universal screening tool
EIMs: Extra-intestinal manifestations
BMI: Body mass index
E1: Proctitis
E2: Left side colitis
E3: Extensive colitis
SD: Standard deviation
IQR: Interquartile range
OR: Odds ratio
CI: Confidence intervals 5-ASA: 5-aminosalicylic acid
RC: Standardized regression coefficient
R^2^: Coefficient of determination
IBS: Irritable bowel syndrome
UK: United Kingdom
IBDQ: Inflammatory bowel disease questionnaire
COVID-19: Coronavirus disease 2019
